# Supplementary material for: Local probe of single phonon dynamics in warm ion crystals
Source: Nat Commun. 2017 Jun 9;8:15712. doi: 10.1038/ncomms15712 (PMC5472711; doi:10.1038/ncomms15712)
Supplement: Supplementary Information — Supplementary Notes and Supplementary References [file ncomms15712-s1.pdf]

SUPPLEMENTARY NOTE 1. RAMSEY SEQUENCE ON THE LOCAL SIDEBAND TRANSITION

The experimental sequence consists of two fast, focussed pulses, which are separated by free evolution, and each of which addressing the blue sideband transition of the outermost ion. The second local pulse is applied with a phase shift of π with respect to the first pulse. In the following we describe the elementary ingredients for the resulting Ramsey scheme.

A. Hamiltonian in the absence of laser pulses

In the absence of laser pulses, the ion chain is described by the Hamiltonian

$$H_{\rm free} = \hbar\omega_0\sigma_+\sigma_- + H_{\rm ph}, \tag{1}$$

where ω_0 is the electronic (carrier) transition frequency and $\sigma_+ = \sigma_-^\dagger = |D\rangle\langle S|$, with $|D\rangle$ and $|S\rangle$ denoting the electronic ground state D and excited state S of the outermost ion in the chain. Since other electronic levels as well as the electronic states of other ions are not populated in the course of the experimental sequence, they can be ignored in the theoretical description. The second term $H_{\rm ph}$ describes the motional states (phonons) of the chain as defined in Eq. (1) of the main manuscript. The phonon Hamiltonian $H_{\rm ph}$ is diagonalized in terms of annihilation operators A_j of the collective modes as

$$H_{\rm ph} = \sum_j \hbar\omega_j A_j^\dagger A_j, \tag{2}$$

where ω_j is the frequency of the jth eigenmode. The basis transformation between collective phonon modes (A_j) and local modes (a_i) is described by a unitary matrix of coefficients β_{ij}, such that

$$a_i = \sum_{\rm j=1}^{N} \beta_{ij} A_j. \tag{3}$$

B. Initial state

After Doppler cooling and optical pumping, the ion chain is initialized in the quantum state:

$$\rho_{\rm in} = |S\rangle\langle S| \otimes \rho_{\rm ph}, \tag{4}$$

where $|S\rangle\langle S|$ denotes the electronic ground state of the first ion and $\rho_{\rm ph}$ represents a thermal state of motion, which is diagonal in the energy eigenbasis [1]

$$\rho_{\rm ph} = \frac{1}{Z_A} e^{-H_{\rm ph}/k_{\rm B}T}, \tag{5}$$

with $Z_A = {\rm Tr}\left(e^{-H_{\rm ph}/k_{\rm B}T}\right)$, Boltzmann constant $k_{\rm B}$ and temperature T.

C. Sideband pulses

The Ramsey pulses in this experiment couple the electronic states of the first ion, $|S\rangle$ and $|D\rangle$, to its motional states via sideband transitions. In the experiment, both Ramsey pulses are focused on the same single ion, while being applied with a pulse duration of $5-13$ µs, which leads to a spectral spread of $2\pi\times(69-178)$kHz. In all of these experiments the spread of the eigenmodes that are involved in the motion of the first ion (with Lamb-Dicke factors of larger than 0.01) is less than $2\pi\times 58$kHz. Therefore, the pulse occurs on a faster time-scale than the phonon tunneling, thereby creating localized phonons at the first ion. In a suitable interaction picture, this local sideband transition is therefore described by the Hamiltonian

$$H = \frac{\hbar\Omega_0}{2}\left(\sigma_+ e^{i(\mathbf{k}_L\cdot\mathbf{x}_1(t)-\Delta\cdot t+\phi)} + \sigma_- e^{-i(\mathbf{k}_L\cdot\mathbf{x}_1(t)-\Delta\cdot t+\phi)}\right), \tag{6}$$

in which $\mathbf{x}_1(t) = U_{\text{free}}(t)^\dagger \mathbf{x}_1 U_{\text{free}}(t)$, where $\mathbf{x}_1$ is the position operator of the first ion, $\mathbf{k}_L$ is the wavevector of the laser, Ω_0 is the Rabi frequency of the driving laser with frequency $\omega_L = \omega_0 + \Delta$ and phase ϕ. Here, Δ is chosen as the frequency of radial oscillations of the first ion along the direction of $\mathbf{k}_L$.

The ion chain is cooled to the Doppler limit to a mean phonon occupation number of $\bar{n} \approx 5$. With a Lamb-Dicke parameter of $\eta \approx 0.05$, we obtain $\eta\sqrt{\bar{n}} \approx 0.11 \ll 1$, hence, we can approximate the Hamiltonian further by assuming the Lamb-Dicke regime [2]. Moreover, we apply a rotating-wave approximation (discarding fast-rotating terms that contain the operators $a_1^\dagger\sigma_+$ and $a_1\sigma_-$) to finally obtain the unitary operator describing the action of the first Ramsey pulse

$$R(\theta,\phi) = \exp\left\{\frac{\theta}{2}\left(e^{i\phi}\sigma_+ a_1^\dagger - e^{-i\phi}\sigma_- a_1\right)\right\}, \tag{7}$$

in which $\theta = \eta\Omega_0\tau$ and τ denotes the pulse duration.

D. Time-evolution under the Ramsey pulse sequence

Let us now describe the evolution of the initial quantum state ρ_{in} under the Ramsey sequence. We first focus on the description of the quantum state after an initial sideband pulse with phase ϕ_1, followed by free evolution for a time t, which leads to the following quantum state:

$$\rho(\theta,\phi_1;t) = U_{\text{free}}(t)R(\theta,\phi_1)\rho_{\text{in}}R^\dagger(\theta,\phi_1)U^\dagger_{\text{free}}(t). \tag{8}$$

The free evolution is described by $U_{\text{free}}(t) = \exp(-iH_{\text{free}}t)$, where H_{free} was introduced in Eq. (1). Since $[H_{\text{free}},\rho_{\text{in}}] = 0$, we can rewrite the above expression as

$$\rho(\theta,\phi_1;t) = R(\theta,\phi_1;-t)\rho_{\text{in}}R^\dagger(\theta,\phi_1,-t), \tag{9}$$

where $R(\theta, \phi_i; -t) = U_{\text{free}}(t) R(\theta, \phi_i) U_{\text{free}}^{\dagger}(t)$. We further express $R(\theta, \phi; -t)$ in terms of collective modes,

$$R(\theta, \phi; -t) = \exp\left(-i \sum_{i=1}^{N} \omega_i A_i^{\dagger} A_i t\right) \exp\left\{\frac{\theta}{2}\left(e^{i(\phi-\omega_0 t)}\sigma_+ \sum_{j=1}^{N} \beta_{1j}^* A_j^{\dagger} - e^{-i(\phi-\omega_0 t)}\sigma_- \sum_{j=1}^{N} \beta_{1j} A_j\right)\right\}$$
$$\times \exp\left(i \sum_{i=1}^{N} \omega_i A_i^{\dagger} A_i t\right).$$

The bosonic commutation relations of the collective mode operators lead to interaction-picture operators of the form $e^{-i\omega_j A_j^{\dagger} A_j t} f(A_j^{\dagger}, A_j) e^{i\omega_j A_j^{\dagger} A_j t} = f(A_j^{\dagger} e^{-i\omega_j t}, A_j e^{i\omega_j t})$, where $f(A_j^{\dagger}, A_j)$ is some function of the collective mode operators $A_j^{\dagger}$ and A_j. We write

$$R(\theta, \phi; -t) = \exp\left(\frac{\theta}{2} u(\phi, -t)\right), \tag{10}$$

where $u(\phi, -t) = e^{i(\phi-\omega_0 t)}\sigma_+ A(t)^{\dagger} - e^{-(i\phi-\omega_0 t)}\sigma_- A(t)$. We have further introduced

$$A^{\dagger}(t) = a_1^{\dagger}(-t) = \sum_{j=1}^{N} \beta_{1j}^* A_j^{\dagger} e^{-i\omega_j t} = \sum_{k=1}^{N} \gamma_k(t) a_k^{\dagger}, \tag{11}$$

with

$$\gamma_k(t) = \sum_{j=1}^{N} \beta_{1j}^* \beta_{kj} e^{-i\omega_j t}. \tag{12}$$

Finally, after application of the second Ramsey pulse (with phase ϕ_2 and same duration $\theta/2$ as the first one), the density matrix is given by

$$\begin{aligned}\rho_{\text{f}}(\theta, \phi_1, \phi_2; t) &= R(\theta, \phi_2; 0)\rho(\theta, \phi_1; t) R^{\dagger}(\theta, \phi_2; 0),\\ &= R(\theta, \phi_2; 0) R(\theta, \phi_1; -t)\rho_{\text{in}} R^{\dagger}(\theta, \phi_1; -t) R^{\dagger}(\theta, \phi_2; 0).\end{aligned} \tag{13}$$

E. Visibility

In the experiment, we measure the population of the $|D\rangle$ state, which is given by

$$P_D(\theta, \phi_1, \phi_2; t) = \text{Tr}\left[P_D \rho(\theta, \phi_1, \phi_2; t)\right], \tag{14}$$

where Tr denotes the trace, $P_D = |D\rangle\langle D| \otimes \mathbb{I}_N$, and $\mathbb{I}_N$ is the identity operator on the Fock space of the N bosonic modes. With the final state $\rho_{\text{f}}(\theta, \phi_1, \phi_2; t)$, as defined in Eq. (13), we obtain

$$P_D(\theta, \phi_1, \phi_2; t) = \text{Tr}\left[P_D \exp\left(\frac{\theta}{2} u(\phi_2, 0)\right) \exp\left(\frac{\theta}{2} u(\phi_1, -t)\right) \rho_{\text{in}} \exp\left(-\frac{\theta}{2} u(\phi_1, -t)\right) \exp\left(-\frac{\theta}{2} u(\phi_2, 0)\right)\right]. \tag{15}$$

This result is valid in the Lamb-Dicke regime for arbitrary pulse duration $\theta/2$. To find an analytical expression for the Ramsey fringe visibility we approximate this expression further, based on experimentally motivated assumptions.

In the experiment, the normal mode frequencies are of the order of $\omega_j \approx 2\pi \times 2\,\mathrm{MHz}$, while the spread of normal modes is given by $\Delta\omega \lesssim 2\pi \times 100\,\mathrm{kHz}$. Therefore, $\Delta\omega/\omega_j \approx 0.05$, which implies that the average number of phonons in normal modes $\bar{n}_j = \langle A_j^\dagger A_j \rangle_{\rho_{\mathrm{ph}}} = \mathrm{Tr}\{A_j^\dagger A_j \rho_{\mathrm{ph}}\}$ are approximately equal. Hence, we assume that the initial state describes an incoherent, homogeneous phonon distribution among the collective modes, i.e.,

$$\left\langle A_j^\dagger A_k \right\rangle_{\rho_{\mathrm{ph}}} \approx \bar{n}\delta_{jk}. \tag{16}$$

Furthermore, we expand $R(\theta, \phi; -t)$ up to second order in the pulse duration $\theta/2$. In all of our measurements, $\theta/2 \lesssim 0.32$. We obtain

$$P_D(\theta, \phi_1, \phi_2; t) = \left(\frac{\theta}{2}\right)^2 (\bar{n}+1) \sum_{j,k,l=1}^{N} \left(e^{i\phi_2}\gamma_j(t) + e^{i\phi_1}\delta_{j1}\right)\left(e^{-i\phi_2}\gamma_j^*(t) + e^{-i\phi_1}\delta_{j1}\right)\beta_{jk}^*\beta_{lk} + O(\theta^4), \tag{17}$$

where $\bar{n}$ is the average phonon occupation number per normal modes. Using $\sum_{j=1}^{N} |\gamma_j(t)|^2 = 1$ and $\Delta\phi = \phi_1 - \phi_2$, the expression above can be simplified to

$$P_D(\theta, \phi_1, \phi_2; t) = \left(\frac{\theta}{2}\right)^2 (\bar{n}+1)\left(2 + e^{-i(\Delta\phi)}\gamma_1(t) + e^{i(\Delta\phi)}\gamma_1^*(t)\right) + O(\theta^4). \tag{18}$$

The fringe visibility is now given by

$$v(t) = \frac{\max(P_D(t)) - \min(P_D(t))}{\max(P_D(t)) + \min(P_D(t))} \approx |\gamma_1(t)|, \tag{19}$$

where the maximum and minimum are taken by optimizing over the phase difference $\Delta\phi$ between the two Ramsey pulses. The final result is obtained by writing $\gamma_1(t) = |\gamma_1(t)|e^{i\varphi(t)}$ and realizing that $\max(P_D(t))$ and $\min(P_D(t))$ are attained at $\Delta\phi = \varphi(t)$ and $\Delta\phi = \varphi(t) + \pi$, respectively. The function $\gamma_1(t)$ was introduced in Eq. (12). The result (19) was used to predict the time evolution of the visibility in Figures 3 and 4 of the main manuscript.

SUPPLEMENTARY NOTE 2. TWO-POINT CORRELATION FUNCTION

The above Ramsey scheme can be interpreted as a method to extract the two-point auto-correlation function of the phonons by means of spin measurements. To see this, consider the first ion's phonon annihilation operator in the Heisenberg picture, which using Eq. (3) reads,

$$a_1(t) = e^{+iH_{\mathrm{free}}t} a_1 e^{-iH_{\mathrm{free}}t} = \sum_j \beta_{1j} A_j e^{-i\omega_j t}. \tag{20}$$

With this, the two-point auto-correlation function of the phonons is given by

$$\langle a_1(t) a_1^\dagger(0)\rangle = \sum_{jk} \beta_{1j}\beta_{1k}^* e^{-i\omega_j t}\langle A_j A_k^\dagger\rangle. \tag{21}$$

With the assumption of an initial phonon state that is diagonal in the collective mode basis, and whose population is independent of the mode index j, c.f. Eq. (16), we obtain

$$\begin{aligned}\langle a_1(t)a_1^\dagger(0)\rangle &= \sum_j \beta_{1j}\beta_{1j}^* e^{-i\omega_j t}\langle A_j A_j^\dagger\rangle \\ &= (\bar{n}+1)\gamma_1(t),\end{aligned} \tag{22}$$

where, again, $\bar{n} = \langle A_j^\dagger A_j\rangle$. Thus, the experimental scheme may be seen as a local method to directly determine the modulus of the two-point correlations functions of the phonon chain.

Based on this argument, we expect that suitable extensions of the scheme to protocols consisting of more than two pulses are in fact capable of extracting also higher-order phonon auto-correlation functions. This way, the methods of non-linear spectroscopy, which are typically employed to study dynamical and spectral features of molecular aggregates and semiconductors, become accessible to probe this many-body system. This opens up a powerful way of analyzing complex interacting quantum systems, especially if combined with the single-site addressability that is unique to quantum optical systems [4–7].

SUPPLEMENTARY NOTE 3. MONITORING OF SPIN-PHONON DISCORD DYNAMICS

In this section, we show that the Ramsey signal discussed above reveals the dynamics of discord-type correlations between the electronic and motional degree of freedom of the first ion. To this end, we revisit the density matrix $\rho(\theta,\phi_1;t)$ of the total system after the first sideband pulse and after free evolution of the chain (duration t), which was previously introduced in Eq. (9). Within second order in $\theta/2$ the density matrix is given by

$$\begin{aligned}\rho(\theta,\phi_1;t) = {}& |0\rangle\langle 0|\otimes\Big[\rho_{\rm ph} - \frac{\theta^2}{8}\Big(A(t)A^\dagger(t)\rho_{\rm ph}^{(1)} + \rho_{\rm ph}A(t)A^\dagger(t)\Big)\Big] \\ &+ |1\rangle\langle 1|\otimes\frac{\theta^2}{4}A^\dagger(t)\rho_{\rm ph}A(t) \\ &+ \frac{\theta}{2}\Big(|1\rangle\langle 0|\otimes e^{i\phi_1}A^\dagger(t)\rho_{\rm ph} + |0\rangle\langle 1|\otimes e^{-i\phi_1}\rho_{\rm ph}A(t)\Big).\end{aligned} \tag{23}$$

where $A(t)$ was introduced in Eq. (11). Taking the trace over the local phonons $i = 2,\dots,N$ one finds the density matrix describing the combined quantum state of the electronic and the motional degrees of freedom of the first ion:

$$\begin{aligned}\rho^{(1)}(t,\tau_1,\phi_1) &= {\rm Tr}_{[2,N]}\,\rho(t,\tau_1,\phi_1) \\ &= |0\rangle\langle 0|\otimes Y(t) + |1\rangle\langle 1|\otimes Z(t) \\ &\quad + \frac{\theta}{2}\Big(|1\rangle\langle 0|\otimes e^{i\phi_1}\gamma_1(t)a_1^\dagger\rho_{\rm ph}^{(1)} + |0\rangle\langle 1|\otimes e^{-i\phi_1}\gamma_1^*(t)\rho_{\rm ph}^{(1)}a_1\Big).\end{aligned} \tag{24}$$

Here, $\rho_{\rm ph}^{(1)} = {\rm Tr}_{[2,N]}\,\rho_{\rm ph}$ is the reduced state of the motional degree of freedom of the first ion, while $Y(t)$ and $Z(t)$ are certain operators acting on the corresponding state space. In order to derive (24) from (23) we have used the relation ${\rm Tr}_{[2,N]}\{\rho_{\rm ph}a_k\} = \delta_{k1}\rho_{\rm ph}^{(1)}a_1$.

With the help of expression (24) we can easily determine the quantum discord between the electronic and the motional degrees of freedom of the first ion. A simple way to estimate the discord-type correlations is obtained from the dephasing-induced disturbance [8–10], given by

$$D(t) = \frac{1}{2}\|\rho^{(1)}(t,\tau_1,\phi_1) - \rho_{\rm deph}^{(1)}(t,\tau_1,\phi_1)\|, \tag{25}$$

where $\|\cdot\|$ denotes the trace norm and $\rho_{\rm deph}^{(1)}(t,\tau_1,\phi_1)$ is the state obtained from the state $\rho^{(1)}(t,\tau_1,\phi_1)$ after dephasing in its eigenbasis:

$$\rho_{\rm deph}^{(1)}(t,\tau_1,\phi_1) = |0\rangle\langle 0|\otimes Y(t) + |1\rangle\langle 1|\otimes Z(t). \tag{26}$$

Thus, we have $D(t) = \frac{1}{2}\|X(t)\|$, where

$$\begin{aligned} X(t) &\equiv \rho^{(1)}(t,\tau_1,\phi_1) - \rho_{\rm deph}^{(1)}(t,\tau_1,\phi_1) \\ &= \frac{\theta}{2}\Big(|1\rangle\langle 0|\otimes e^{i\phi_1}\gamma_1(t)a_1^\dagger\rho_{\rm ph}^{(1)} + |0\rangle\langle 1|\otimes e^{-i\phi_1}\gamma_1^*(t)\rho_{\rm ph}^{(1)}a_1\Big). \end{aligned}$$

To evaluate this expression analytically, we realize another approximation. In particular, we consider the local phonon state to be of the form

$$\rho_{\rm ph}^{(1)} = \sum_{n_1=0}^{\infty} p_{n_1}|n_1\rangle\langle n_1|. \tag{27}$$

A detailed discussion of this approximation will be given at the end of this section. Using this, one finds that the eigenvalues of $X(t)$ are given by

$$\pm\frac{\theta}{2}|\gamma_1(t)|p_{n_1}\sqrt{n_1+1}, \qquad n_1 = 0,1,2,\ldots, \tag{28}$$

which yields:

$$\begin{aligned} D(t) = \frac{1}{2}\|X(t)\| &= \sum_{n_1=0}^{\infty}\frac{\theta}{2}|\gamma_1(t)|p_{n_1}\sqrt{n_1+1} \\ &= \frac{\theta}{2}\Big\langle\sqrt{n_1+1}\Big\rangle|\gamma_1(t)|. \end{aligned} \tag{29}$$

This shows that the discord-type quantum correlations of the degrees of freedom of the first ion are proportional to the visibility $|\gamma_1(t)|$. The decay and revival of the visibility observed in the experiment thus correspond to a loss and gain of the quantum correlations between the electronic and motional degrees of freedom of the first ion of the chain.

We can further combine Eqs. (22) and (29), leading to:

$$D(t) = \frac{\theta}{2}\frac{\left\langle \sqrt{n_1+1}\right\rangle}{\bar{n}+1}\left|\langle a_1(t)a_1^\dagger(0)\rangle\right| \approx \frac{\theta}{2}\frac{\left|\langle a_1(t)a_1^\dagger(0)\rangle\right|}{\sqrt{\bar{n}+1}}. \tag{30}$$

The measure of discord-type quantum correlations is thus directly proportional to the modulus of the two-point correlation function of the motional amplitude of the first ion. This result is valid for purturbatively small values of $\frac{\theta}{2}$. For the special case of $\pi/2$ pulses as applies to present experiments,

$$D(t) \approx \frac{\pi}{4}\frac{\left|\langle a_1(t)a_1^\dagger(0)\rangle\right|}{\bar{n}+1}, \tag{31}$$

in which we replaced $\theta\left\langle \sqrt{n+1}\right\rangle$ with $\pi/2$ indicating a Ramsey $\pi/2$-pulse from equation (7). In this case, the perturbation parameter is $\frac{\theta}{2} \approx 0.3$. The equation above leads to

$$D(t) \approx \frac{\pi}{4}v(t), \tag{32}$$

which indicates a direct proportionality between discord and visibility, and thereby proves visibility as a quantitative measure for discord-type quantum correlations in the chain.

To determine the coherences and the discord of the state of the electronic and the motional degrees of freedom of the first ion we have used the additional assumption that the phonon state (5) may be replaced by an equilibrium state of the local modes a_j:

$$\rho_{\rm ph}^a = \frac{1}{Z_a}\exp\left[-\beta\sum_j \hbar\omega_j a_j^\dagger a_j\right]. \tag{33}$$

Averages taken with $\rho_{\rm ph}^A$ are denoted by $\langle ...\rangle_A$ and averages with $\rho_{\rm ph}^a$ are denoted by $\langle ...\rangle_a$. To justify the replacement $\rho_{\rm ph}^A \to \rho_{\rm ph}^a$ we require that all first and second moments of A_j determined by $\rho_{\rm ph}^A$ and $\rho_{\rm ph}^a$ are identical. It is clear that we have the following exact relations:

$$\langle A_i\rangle_A = \langle A_i\rangle_a = 0, \tag{34}$$

$$\langle A_i A_j\rangle_A = \langle A_i A_j\rangle_a = 0. \tag{35}$$

Thus, we only have to demand that also

$$\langle A_i^\dagger A_j\rangle_A = \langle A_i^\dagger A_j\rangle_a \tag{36}$$

holds, which yields the condition

$$\delta_{ij}\bar{n}_j = \sum_k \beta_{ki}\beta_{kj}^*\bar{n}_k. \tag{37}$$

By condition (16) this becomes

$$\delta_{ij}\bar{n} = \sum_k \beta_{ki}\beta^*_{kj}\bar{n}, \tag{38}$$

which is obviously satisfied since the matrix (β_{ij}) is unitary.

SUPPLEMENTARY REFERENCES

[1] Morigi, G. & Eschner, J. Is an ion string laser-cooled like a single ion? *J. Phys. B* **36**, 1041 (2003).

[2] Leibfried, D., Blatt, R., Monroe, C. & Wineland, D. Quantum dynamics of single trapped ions. *Reviews of Modern Physics* **75**, 281–324 (2003).

[3] Mukamel, S. Principles of Nonlinear Optics and Spectroscopy. *Oxford Univ. Press, New York* (1995).

[4] Gessner, M., Schlawin, F., Häffner, H., Mukamel, S. & Buchleitner, A. Nonlinear spectroscopy of controllable many-body quantum systems. *New Journal of Physics* **16** 092001 (2014).

[5] Schlawin, F., Gessner, M., Mukamel, S. & Buchleitner, A. Nonlinear spectroscopy of trapped ions. *Physical Review A* **90**, 023603 (2014).

[6] Gessner, M., Schlawin, F., & Buchleitner, A. Probing polariton dynamics in trapped ions with phase-coherent two-dimensional spectroscopy. *Chem. Phys.* **142**, 212439 (2015).

[7] Lemmer, A., Cormick, C, Schmiegelow, C.T., Schmidt-Kaler, F., & Plenio, M. B. Two-Dimensional Spectroscopy for the Study of Ion Coulomb Crystals. *Phys. Rev. Lett.* **114**, 073001 (2015).

[8] Gessner, M. & Breuer, H.-P. Detecting Nonclassical System-Environment Correlations by Local Operations. *Phys. Rev. Lett.* **107**, 180402 (2011).

[9] Gessner, M. & Breuer, H.-P. Local witness for bipartite quantum discord. *Phys. Rev. A* **87**, 042107 (2013).

[10] Gessner, M. Dynamics and Characterization of Composite Quantum Systems. *PhD Thesis, Albert-Ludwigs-Universität Freiburg*, (2015).
